# Predicting the number of COVID-19 infections and deaths in USA

**DOI:** 10.1186/s12992-022-00827-3

**Published:** 2022-03-28

**Authors:** Amarachukwu Felix Ebubeogu, Chamberline Ekene Ozigbu, Kholoud Maswadi, Azizi Seixas, Paulinus Ofem, Donaldson F. Conserve

**Affiliations:** 1 Department of Software Engineering, University of Malaya, Kuala Lumpur, 50603 Malaysia; 2 Department of Health Services Policy and Management, Arnold School of Public, Health, Columbia, 29208 SC United States; 3 Department of Prevention and Community Health, Milken Institute School of Public Health, The George Washington University, Washington, 20052 United States; 4 Department of Psychiatry and Behavioral Sciences, The University of Miami Miller School of Medicine, Miami, 33136 FL United States; 5 Department of Software Engineering, University of Malaya, Kuala Lumpur, 50603 Malaysia; 6 Department of Management Information Systems, Jazan University, Jazan, 45142 Saudi Arabia

**Keywords:** 2019 novel coronavirus, COVID-19, SARS-CoV-2, Infection entropy, Infection density

## Abstract

**Background:**

Uncertainties surrounding the 2019 novel coronavirus (COVID-19) remain a major global health challenge and requires attention. Researchers and medical experts have made remarkable efforts to reduce the number of cases and prevent future outbreaks through vaccines and other measures. However, there is little evidence on how severe acute respiratory syndrome coronavirus 2 (SARS-CoV-2) infection entropy can be applied in predicting the possible number of infections and deaths. In addition, more studies on how the COVID-19 infection density contributes to the rise in infections are needed. This study demonstrates how the SARS-COV-2 daily infection entropy can be applied in predicting the number of infections within a given period. In addition, the infection density within a given population attributes to an increase in the number of COVID-19 cases and, consequently, the new variants.

**Results:**

Using the COVID-19 initial data reported by Johns Hopkins University, World Health Organization (WHO) and Global Initiative on Sharing All Influenza Data (GISAID), the result shows that the original SAR-COV-2 strain has *R*_0_<1 with an initial infection growth rate entropy of 9.11 *bits* for the United States (U.S.). At close proximity, the average infection time for an infected individual to infect others within a susceptible population is approximately 7 minutes. Assuming no vaccines were available, in the U.S., the number of infections could range between 41,220,199 and 82,440,398 in late March 2022 with approximately, 1,211,036 deaths. However, with the available vaccines, nearly 48 Million COVID-19 cases and 706, 437 deaths have been prevented.

**Conclusion:**

The proposed technique will contribute to the ongoing investigation of the COVID-19 pandemic and a blueprint to address the uncertainties surrounding the pandemic.

## Introduction

The COVID-19 outbreak has remained a universal health concern that requires urgent attention. Notably, Scientists have pointed out that COVID-19 is the newest coronavirus species with public health emergency [1–3]. Although several studies have been carried out aimed to provide useful information regarding the coronavirus pandemic [4–7], studies are still ongoing to uncover the root cause of the pandemic as well as solutions to address the outbreak. In December 2019, there were clusters of pneumonia cases in China. Later, investigations discovered that an unknown virus caused such clusters of pneumonia [8, 9]. The unknown virus is currently called the 2019 novel coronavirus. Coronaviruses are a large group of viruses that consist of core genetic materials surrounded by specific protein spikes [10, 11]. Despite their unique nature, there are different types of coronaviruses that cause respiratory symptoms. These symptoms may range from the common cold to pneumonia, as in China’s case, where it was first identified. These symptoms may be mild in most cases, whereas some cases are severe. For instance, fever, cough, and shortness of breath may be signs of mild symptoms. On the other hand, pneumonia, kidney failure, and death characterize severe cases. However, some kinds of coronaviruses are responsible for severe cases, such as severe acute respiratory syndrome coronavirus (SARS-CoV), first discovered in China in 2002–2003 [12–14]. Another type of coronavirus that can also cause severe health damage is the Middle East respiratory syndrome-related coronavirus (MERS-CoV), identified in the Kingdom of Saudi Arabia in 2012 [15, 16].

Despite the symptoms of MERS-CoV, which include sore throat, headache, fever, mild cough, tiredness, runny nose and diarrhea, its transmission among humans appears to be less harmful [17]. SARS-CoV-2 is characterized by more contagious variants [18–21]. In terms of structure, the S proteins of SARS-CoV and SARS-CoV-2 are similar [22, 23]. In terms of *R*_0_, a daily reproduction number of 2.68 was reported by Wu et al. for SARS-CoV-2 [22], equivalent to the reports by both the WHO and the Chinese Center for Disease Control [24, 25]. A previous study reported an actual *R*_0_ value between 2.0 and 2.5 for SARS-CoV-2, which remains disputed [26]. However, the disputed *R*_0_ values for SARS-CoV-2 are lower than the 1.7 and 1.9 *R*_0_ for SARS and *R*_0_ <1 for MERS, respectively, [26]. In addition, the *R*_0_ values for SARS-CoV-2 have been estimated to range between 2.24 and 3.58 [27], while another study reported a range between 2.0 and 5.0 for SARS [28]. Similarly, certain predictive models have suggested *R*_0_ values of 3.8 (95% CI, 3.6–4.0) [29] and 3.11 (95% CI, 2.39–4.13) for SARS-CoV-2 [30]. For SARS, the *R*_0_ was estimated to be approximately 3.0 if adequate control measures were not in place [31]. A high value of 5.8 (confidence interval: 4.7–7.3) for SARS-CoV-2 was reported for the U.S., and a range between 3.6 and 6.1 was reported for some countries in Europe [32]. Notably, a group of researchers reported a higher *R*_0_ value of 6.47 for SARS-CoV-2 [33]. These high values of *R*_0_ indicate that the SARS-CoV-2 virus has the ability to rapidly mutate and spread [2, 34].

Notably, COVID-19 poses a health threat and an economic threat across the globe [35]. Obviously, the gross domestic product (GDP) of almost all the affected countries has dropped tremendously, and as such, goods and services are affected along the supply chains. Besides, millions of schools from kindergarten to institutions of higher learning remain closed. Millions of both private and public companies, as well as their respective employers and employees, are under lockdown. Consequently, millions of workers have lost their jobs as a result of this pandemic.

Notwithstanding the remarkable achievements of the existing studies on providing useful information regarding the COVID-19 pandemic, the following gaps exist in the literature: (1) how the daily infection density attributes to increase in number of cases, (2) how daily infection entropy can be applied in predicting the number of cases and deaths and (3) the average time it may take for an infected individual to infect a susceptible population in close proximity.

Therefore, more studies are needed to support medical expert investigations as well as in their decision-making processes to uncover novel preventive measures to complement the available vaccines for the 2019 novel coronavirus. Hence, to better understand and characterize the initial behaviour of the virus, the current study aggregates the number of the initial reported COVID-19 cases before the emergence of the new variants and the corresponding numbers of deaths for March 2020 in the U.S. Based on the relevant data obtained, there is an indication that the daily infection entropy can be applied in predicting the likelihood of infection at a given period. In addition, there is a relationship between the daily infection density and the time of infection. The study therefore hypothesize that, with the emergence of the new variants, average time of infection in close proximity is <7 minutes.

Finally, using the initial COVID-19 dataset in the U.S., this study shows how SARS-CoV-2 infection entropy can be applied in predicting the possible number of infections and deaths within a given population. Such an approach can be applicable to other disease outbreaks.

## Materials and methods

In this section, we present the measures applied to evaluate the current study. First, infection density (*β*) is defined as the ratio of the number of infections at constant population. In this study, the unit of measurement for *β* is number per population. Infection acceleration is defined as the change in daily infection velocity (*υ*_*i*_) over time. Note, the infection acceleration, gain in virus momentum, rate of infection, and increase in the number of infections represent the same measure. These measures indicate the change in behavior of the original SARS-COV-2 virus strain that may result in transmissible new variants. Other metrics include: entropy applied to determine the uncertainties in infections and deaths. The entropy is measured in *bits* [36].

**Fig. 1:**
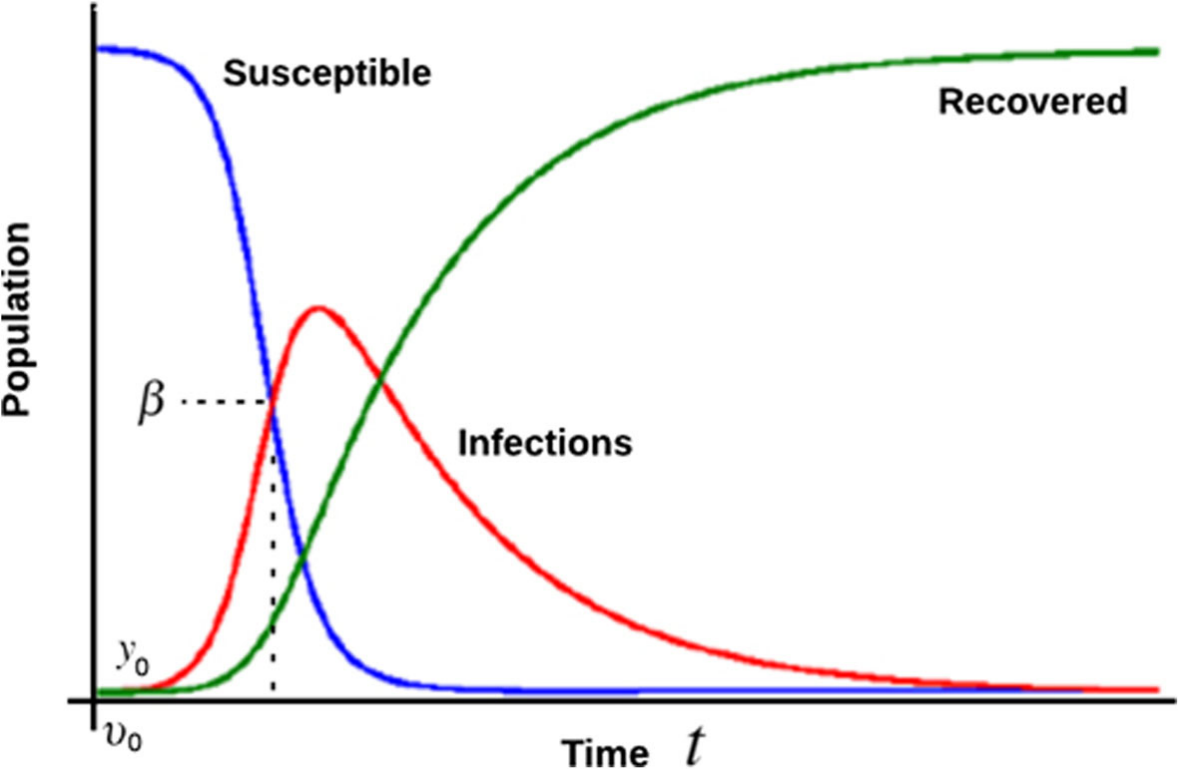
SIR Model with infection density. Here *β* represents the infection density, *υ*_0_ represents the initial infection velocity and *y*_0_ represents the initial phase (position) of infection
- Rate of infection in terms of infection acceleration influences the number of infections within a given population.
- Increase in the number of infections is due to the rate of daily spread *β*.
- The rate of daily spread and the infection density over a period depends on the rate at which the susceptible population is exposed to the virus and, consequently, gets infected. Hence, the rate of daily spread is equivalent to daily infection density.
- A given population can easily be infected at close proximity with an infected individual.

In Fig. 1, susceptible *S*, refers to the population who may be vulnerable to infection, infections (infected) *I*, are those who are infected by COVID-19, the recovered *R* is referred to those who with no symptom as a result of vaccines, antibodies or immune as well as those who may have died as a result of COVID-19. Considering the Susceptible Infections and Recovered (SIR) Model, we make the following assumptions:

- A constant population with an increasing the number of infections.

The variables within the SIR model can be represented mathematically [37–39] as follows:

For the susceptible we have:

1 $$ \frac {dS}{dt} = -\alpha SI  $$

where*S* = the susceptible,*I* = infected, *α* = daily reproduction rate and *t* = timeAssuming a decreasing number of susceptible at constant population, as *S* transits to *I* due to the rate of infections, the value of *S* decreases over time. Hence the value of *α* will remain (-ve) which shows a decrease in the number of susceptible [37–39].

For the infected we have:

2 $$ \frac {dI}{dt} = \alpha SI - \beta I  $$

where*S* = the susceptible,*I* = infected, *α* = daily reproduction rate, *β* = the rate of daily spread ≡ daily infection density and*t* = time

Assuming an increase in the number of infections due to high contact rate. Hence the value of *αSI* remains (+ve).

However, if the ratio of daily reproduction rate and the rate of daily spread is greater than 1, (i.e., $\frac {\alpha }{\beta } >$1), there is every possibility that the infection will rapidly spread. On the other hand, if $\frac {\alpha }{\beta } <$1, there could be spread with no exponential growth [37–39].

For the recovered we have:

3 $$ \frac {dR}{dt} = \beta I  $$

This indicates that those who have recovered from the infection due to antibodies or vaccines and may not be reinfected or even those who died. These values are excluded from the infection over time. Hence the −*βI* in $\frac {dI}{dt}$ is regained as +*βI* as presented in Equation 3.where*I* = infected,*R* = recovered, *β* = the rate of daily spread ≡ daily infection density and*t* = time

### Computation of *α*,*β* and *R*_0_

Note, the daily reproduction rate is computed using the expression

$$\alpha = \frac{No.\; of \;infections\; - \;No. \;of\; deaths}{population\;size} \times 100 $$

$\frac {\alpha }{\beta }= \;R_{0}$,which represents the basic reproduction number or the basic reproductive ratio, can be assumed to be the expected number of cases resulting from a single infection within a population where all individuals are susceptible. A higher value of *R*_0_ means that the infection would be easily transmitted. *R*_0_<1 means that the new cases will decrease over time, and ultimately, the outbreak will end on its own. *R*_0_=1 means the cases may be stable over time, whereas *R*_0_>1 indicates the virus may be autonomous, mutate into new variants, rapidly spread, and requires stringent and efficient control measures.

Looking back in March 2020, the virus’s initial behavior shows how the infections will be over time with a high value of *R*_0_. Indicating a significant increase in positivity rate and new infection clusters, consequently increasing hospitalizations and deaths.

This implies that the daily infection density characterizes the daily reproduction rate of the SARS-CoV-2 virus. Hence,

$$ {f} \;(number\; of\; infections)\; \;\;\Rightarrow\;\; \;\beta $$

The *β* is influenced by the number of infections within the population, which typically depends on the rate of infections. The rate of infection in this context represents the acceleration of infection. At a constant population, an increase in infection rate will lead to an increase in infection density. Consequently, if the rate (acceleration of infection) decreases, it will lead to a decrease in infection density and a decrease in the rate of daily spread.

Thus,

$$ {f} \;(infection \;acceleration)\; \;\;\Rightarrow\;\; \;{number\; of\; infections} $$

The above expression implies that the increase in the number of infections is a function of infection acceleration; hence, increasing the *β*.

Therefore,

$$ {f} \;(infection \;acceleration)\; \;\;\Rightarrow\;\; \;\beta $$

The infection acceleration is defined as the change in infection velocity over the change in time as follows:

4 $$ {}infection\ acceleration\! =\! \frac {change\ in \ infection\ velocity\ (\upsilon_{i})} { \;time\;}  $$

At a constant population, the amount of infections and *β* within a population change as a result of infection acceleration. Thus,

5 $$ \beta =\frac {number\;of\;infections} {const.\; population\;} \equiv \frac {f(infection \; \;acceleration)} {const.\; population\;}  $$

The *β*, which depends on the infection acceleration at a contact population, can be expressed as shown in Eqs. (6) and (7):

6 $$ \beta =\frac {f(infection\;acceleration)} {const.\; population\;}  $$

7 $$ \beta=\frac {change\; in \;infection\; velocity\;(\upsilon_{i})}{ time}  $$

8 $$ \upsilon_{i}=\beta \times time  $$

where *β* = daily infection density and *v*_*i*_ = change in infection velocity.

By integrating the *υ*_*i*_ over time, we obtain

9 $$ \int \upsilon_{i}dt =\int \beta dt  $$

10 $$ \beta \int dt=\beta t+c  $$

The initial *υ*_*i*_ before the outbreak equal to zero (i.e., *υ*_*i*_ = 0). Therefore, replacing *c* with this initial infection velocity, we have

11 $$ \beta \int dt=\beta t+ (\upsilon_{i} = 0)  $$

To determine the stage of infection in terms of its position (*y*_*i*_) within the susceptible population over time, we apply the velocity relationship [40], expressed as

12 $$ \upsilon =\frac {change\;in\; position(y_{i})} {time (t)}  $$

where *v* = infection velocity.

Hence, to obtain the target infection stage *y*_*i*_ with respect to *t*, we integrate accordingly *y*_*i*_ with respect to *t*, we have

13 $$ y_{i}(t) =\int \upsilon dt  $$

Under the assumption that (*υ*=*υ*_*i*_), by integrating the infection velocity over time, similar to Equation (9), yields

14 $$ \int \upsilon dt = \int \beta t+\upsilon_{0}dt  $$

As we hope to determine the infection stage in terms of the position within a population, there is also a need to estimate the time these infections occur. From Equation (14) above, going further, we can determine *t* of infection as

15 $$ \int \beta t+ \upsilon_{0}dt = \int \beta tdt+\int \upsilon_{0}dt  $$

16 $$ = \beta \int tdt+\int \upsilon_{0}dt  $$

17 $$ = \beta \left(\frac {1} {2}t^{2}\right) +\upsilon_{0}t+c  $$

Here, *c* which represents the initial infection stage before the outbreak is equal to zero (i.e., *c*=*y*_*i*_=0). In addition, at this early stage, the amount of infections as well as the infection velocity are both equal to zero.

18 $$ y_{i}(t)= \beta \left(\frac{1} {2}t^{2}\right) + \upsilon_{0}t + (y_{i} = 0)  $$

Hence, if *υ* = *υ*_*i*_, we can now rewrite the above Equation as;

19 $$ y_{i}(t)= \beta \left(\frac{1} {2}t^{2}\right) + (\upsilon_{i}= 0)t + (y_{i} = 0)  $$

20 $$ y_{i}(t)= \beta \left(\frac{1} {2}t^{2}\right)  $$

To estimate the infection time (i.e., the average time it take for an infected individual to transmit the virus daily to a susceptible population at proximity), from the resulting Equation (19), we have

21 $$ t=\sqrt {\frac {2y_{i}} {\beta}}  $$

where *y*_*i*_ represents the stage of daily infection within the population, *t* represents infection time and *β* is the daily infection density.

#### Entropy

As applied in the current study, entropy can be referred to as the quantity of information uncertainties acquired from the information source measured in *bits* [36]. As the amount of uncertainties surrounding COVID-19 infection varies, the concept of entropy is applied to assess the initial daily infection uncertainties.

Thus, the entropy of an information source *s* on the daily infection growth rate (IGR) can be denoted by *IGR*(*s*). To determine *IGR*(*s*), we apply

22 $$ IGR(s)=\sum_{i=1}^{n}p_{i} \;log_{2}\;\left(\frac{1}{p_{i}}\right)  $$

We can rewrite the above equation as

23 $$ IGR(s)=p_{1} \;log_{2}\;\left(\frac{1}{p_{1}}\right)\;+\;...\;p_{n}\;log_{2}\;\left(\frac{1}{p_{n}}\right)  $$

where *p*_*i*_ represents the probability outcome for daily infection with respect to the uncertainties surrounding the information source, *IGR*(*s*) is the daily infection growth rate and *n* is the number of sources of information.

### Justification for choice of model

Despite remarkable achievements in the fight against the virus, there are still unknown factors surrounding the COVID-19 pandemic. However, the simplicity of our proposed model reveals how important it is to consider all possible parameters that might be responsible for the increase in the number of COVID-19 cases, consequently, the new variants. In addition, it shows how the daily infection density can be modeled via the SIR model as well as an easy to replicate approach. Notably, the current study does not involve any human or animal subjects. This study relied on the COVID-19 data reported by John Hopkins University [41], the World Health Organization [42] and the Global Initiative on Sharing All Influenza Data [43]. These datasets did not indicate the number of hospitalized persons or quarantined individuals but rather a generalized number of cases and deaths, respectively.

### Data collection

The datasets applied in the current study are presented in Table 1. This study utilizes the initial COVID-19 records as reported by John Hopkins University [41], WHO [42] and GISAID [43]. Table 1 presents the number of initially reported cases with respect to the original strain of SARS-CoV-2 virus in the U.S.

**Table 1:** Numbers of reported COVID-19 cases and the corresponding numbers of deaths for March 2020 in the U.S. Here *IGR* represents the infection growth rate, *DGR* represents death growth rate, and *β* represents the infection density measured in (num/reported cases)

March	No. of Reported Cases	Difference in the No. of Cases	IGR	No. of Deaths	Difference in the No. of Deaths	DGR	Entropy of IGR & DGR (*bits*)	Infection Density (*β*)	*R*_0_
1	75	7	0.1	1	0	0	0.12	2.28e-7	0.985
2	100	25	0.33	6	5	5	-11.2	3.04e-7	0.936
3	124	24	0.24	9	3	0.5	0.79	3.76e-7	0.928
4	158	34	0.27	11	2	0.22	0.65	4.80e-7	0.929
5	221	63	0.4	12	1	0.09	0.59	6.71e-7	0.945
6	319	98	0.44	15	3	0.25	0.75	9.68e-7	0.953
7	435	116	0.36	19	4	0.27	0.78	1.32e-6	0.957
8	541	106	0.24	22	3	0.16	0.51	1.64e-6	0.961
9	704	163	0.3	26	4	0.18	0.55	2.14e-6	0.962
10	994	290	0.41	30	4	0.15	0.67	3.02e-6	0.969
11	1,301	307	0.31	38	8	0.27	0.68	3.95e-6	0.971
12	1,630	329	0.25	41	3	0.08	0.41	4.95e-6	0.974
13	2,183	553	0.34	48	7	0.17	0.61	6.63e-6	0.977
14	2,770	587	0.27	57	9	0.19	0.56	8.41e-6	0.979
15	3,613	843	0.3	69	12	0.21	0.64	1.09e-5	0.987
16	4,596	983	0.27	87	18	0.26	0.71	1.39e-5	0.985
17	6,344	1,748	0.38	110	23	0.26	0.78	1.93e-5	0.980
18	9,197	2,853	0.45	150	40	0.36	0.90	2.79e-5	0.984
19	13,779	4,582	0.5	206	56	0.37	0.93	4.18e-5	0.986
20	19,367	5,588	0.41	255	49	0.24	0.74	5.88e-5	0.987
21	24,192	4,825	0.25	301	46	0.18	0.50	7.34e-5	0.988
22	33,592	9,400	0.39	414	113	0.38	0.83	1.02e-4	0.987
23	43,781	10,189	0.3	555	141	0.34	0.73	1.33e-4	0.986
24	54,856	11,075	0.25	780	225	0.41	0.73	1.67e-4	0.983
25	68,211	13,355	0.24	1027	247	0.32	0.69	2.07e-4	0.985
26	85,435	17,224	0.25	1295	268	0.26	0.64	2.59e-4	0.986
27	104,126	18,691	0.22	1695	400	0.31	0.63	3.16e-4	0.984
28	123,578	19,452	0.19	2220	525	0.31	0.54	3.75e-4	0.982
29	143,491	19,913	0.16	2583	363	0.16	0.44	4.36e-4	0.981
30	163,788	20,297	0.14	3141	558	0.26	0.59	4.97e-4	0.981
31	188,530 (*N*_*ti*_)	24,742	0.15	4053 (*N*_*td*_)	912	0.29	0.56	5.72e-4	0.978
			$\sum = 9.11$			$\sum =12.45$	$\sum =8.05$		

### Accumulated number of infections *I*_*cu*_

The accumulated number of infections represents the possible number of COVID-19 cases over a given period (weeks or months) with respect to the average infection entropy. The accumulated number of infections is calculated as follows:

24 $$ I_{cu(lower limit)}= N_{ti}(m*\left(\sum_{i=1}^{n}p_{i} \;log_{2}\;\left(\frac{1}{p_{i}}\right)\right)  $$

25 $$ I_{cu(upper limit)}= N_{ti}(m*\left(\sum_{i=1}^{n}p_{i} \;log_{2}\;\left(\frac{1}{p_{i}}\right)\right) * (2)  $$

where *I*_*cu*_ = the accumulated number of infections over a given period, *N*_*ti*_ = the total number of infected cases at a given period,*m* = number of months and *p*_*i*_ represents the probability outcome for daily infection with respect to the uncertainties surrounding the information source.

For example, if the total number of infections in the U.S., as of March 31, 2020, is 188,530 cases. To estimate the lower limit of possible number of infections in late March 2022(24 months apart), using Equation (24), we have:

$$ I_{cu(lower\;limit)}= 188530(24(9.11)) = 41,220,199  $$

To estimate the upper limit of infections using Equation (25), we have:

$$ I_{cu(upper\;limit)}= 188530(24(9.11)) *2 = 82,440,398  $$

This number means that in late March 2022, the possible number of infections in the U.S., may be within the range of 41,220,199 and 82,440,398 but can be reduced if the necessary preventive guidelines are followed. Thus, the difference between the upper limit of infection and the possible number of cases prior to vaccine roll-out (i.e., 82,440,398 - 34,350,166 = 48,090,232). This means that nearly 48 Million Americans have been prevented from COVID-19 infections and hospitalization since the vaccine rolled out. Note (34,350,166 is the upper limit of the predicted number of infections prior to the vaccines roll out in February 2021, 10 months apart).

### COVID-19 average death growth rate

The average death growth rate represents the uncertainties in the number of deaths over a specified period. This metric allows us to keep track of the rate of deaths as a result of COVID-19 over time. The average death growth rate will also help us estimate the possible number of future COVID-19 death in the U.S.

### Accumulated number of deaths *D*_*cu*_

The accumulated number of deaths represents the possible number of COVID-19 deaths cases over a given period (weeks or months) with respect to the average death entropy. The accumulated number of deaths is calculated as follows:

26 $$ D_{cu}= N_{td}(m*\left(\sum_{i=1}^{n}p_{i} \;log_{2}\;\left(\frac{1}{p_{i}}\right)\right)  $$

where

*D*_*cu*_ = accumulated number of deaths at given period,

*N*_*td*_ = the total number of deaths at a given period,

*m* = number of months and *p*_*i*_ represents the probability outcome for daily infection with respect to the uncertainties surrounding the information source.

For example, if the number of deaths in the U.S., as of March 31, 2020, is 4053 deaths. To predict the possible number of deaths in late March 2022, using Eq. (26), we have:

$$ D_{cu} = 4053(24(12.45)) = 1,211,036  $$

This means with no vaccines available, in late March 2022, at least 1,211,036 deaths may be reported in the U.S., alone. However, with the available vaccines, nearly 706,437 deaths have been prevented.

Note, (504, 599 is the predicted number of deaths prior to the vaccines roll out in February 2021).

## Results

The current study shows that gain in momentum of COVID-19 is influenced by the number of infections within a given population, consequently resulting in a higher daily *R*_0_. At constant population, again in the momentum of infection will result in a gain in infection density. Therefore, if the growth in momentum decreases, it will result in a lower infection density and a decrease in the ratio of daily reproduction rate and the rate of daily spread, respectively.

Notably, the exponential increase in the number of infections on a daily basis in March 2020, is characterized by high ratio of daily reproduction rate and the rate of daily spread *R*_0_≥0.9 and *R*_0_ <1 for the original SAR-COV-2 virus strain. However,

On the average infection and death growth rates, *IGR* achieved an entropy of 9.11 *bits*, whereas *DGR* achieved an entropy of 12.45 *bits*, as presented in Table 1. These uncertainties in terms of information entropy are the determinants for future forecasts on the possible number of infections and deaths. Thus, assuming no vaccines were available, in the U.S., the number of infections could range between 41,220,199 and 82,440,398, in late March 2022, with approximately, 1,211,036 deaths. However, with vaccine roll-out, approximately 48 million COVID-19 cases and 706,437 deaths have been prevented. Furthermore, the current study shows that it takes approximately 7 minutes on average for an infected individual to infect others within a susceptible population in close proximity. Hence, from the initial characteristics of the SAR-CoV-2 virus, a single person with COVID-19 can infect approximately 9 people within 1 hour and 216 people in a single day in the U.S.

## Discussion

In this study, we demonstrated how the daily reproduction number of SARS-CoV-2 virus can be determined through the infection density within a given susceptible population. In addition, the current study also shows how the information entropy obtained during the early phase of the outbreak in the U.S., can be applied as determinants for predicting the number of infections and deaths. While numerous underlying but unknown factors surrounding the spread of COVID-19 still exist [44], these unknown factors may also avert the reliability of existing models in predicting and monitoring COVID-19 [45–48]. As a result, the SARS-CoV-2 virus continues to gain momentum with high stakes on human lives. Hence, it will be necessary to formulate models that can access the gain in momentum of the SARS-CoV-2 virus, which enables its ability to spread, resulting in multiple new variants [49–52].

As the need for early detection of COVID-19 infections arises, certain predictive models can be helpful in identifying potential cases [53]. For instance, a logistic model was used to predict the total number of infections to be 4 million during the outbreak in the U.S., [54]. Some of the existing models include but are not limited to the susceptible-infectious-susceptible (SIS) model, the susceptible-infected-recovered-deceased (SIRD) model alongside the SIR model, the infectious disease dynamics model and the time-dependent dynamic model previously applied in predicting the outcome of COVID-19 [44, 55–58]. An infectious disease dynamic model (SEIR) model was applied to model and predict the number of COVID-19 cases in Wuhan, China [57]. The results of that study indicate unstable values of daily reproduction rates, which may lead to a continuous increase in cases in Wuhan if public health intervention is not implemented.

A time-dependent dynamic model has been applied as a measure of public health intervention enhancement strategy through self-isolation [58]. The results obtained using the time-dependent dynamic model indicated that the daily reproduction rate of coronavirus has fallen below 1. However, the virus will continue to spread within the susceptible population [58]. On the possible time to transmit the virus from an infected person to a susceptible population, a study further reported that it might take approximately 10 mins for an infected person to produce 6,000 particles of aerosol [51]. These particles may be potentially harmful and may not be seen easily with the human eye [59].

A Markovian stochastic framework has been proposed to analyze both the reproductive ratio and the entropy of COVID-19. These results indicated a significant but steady difference in the COVID-19 reproduction ratio and entropy, respectively, with a clear indication of the uncertainties surrounding the pandemic [60, 61]. It is therefore important to note that information entropy can be useful in differentiating between severe and mild COVID-19 patients [62–64].

However, a variation in daily reproduction ratio may vary from one location to another based on certain parameters, such as the stage of the outbreak (i.e., the rate of infection) [28]. Therefore, it is important to determine the value of the daily reproduction number at every stage of infection [28]. One of the factors responsible for the rapid transmission of COVID-19 is the daily reproduction number. However, it may be challenging to accurately determine the daily reproduction number [65, 66].

A compartmental mathematical model was formulated to predict the evolution of the virus in Cameroon and to analyze the reported cases in Brazil [67, 68]. The results achieved using these compartmental mathematical models indicated that the dynamics of COVID-19 disease are influenced by variations in the value of *R*_0_.

During the early phase of the pandemic, the basic reproduction ratio was estimated to range between 4.02 to 1.51 and 4.22 ±1.69 for the U.S., and some parts of Europe, respectively, indicating a variation in the daily reproduction ratio [69, 70]. This variation in *R*_0_ is characterized by the uncertainties surrounding the COVID-19 pandemic [26]. Hence, there is an indication that the *R*_0_ for SARS-CoV-2 is higher than the *R*_0_ for both SARS and MERS. As indicated earlier, previous study reported a range between 1.7 and 1.9 as the value of *R*_0_ for SARS and *R*_0_ <1 for MERS while the *R*_0_ for SARS-CoV-2 ranges between 2.0 and 2.5 [26].

This study also recognized some other approaches applied in uncovering useful information regarding the pandemic. For example, a study aims to properly identify critical information in an unprecedented situation such as this outbreak via a natural language processing approach to classify COVID-19-related information [71]. Such an approach enabled the extraction of certain predictor variables that can be used in predicting the amount of reposted information regarding COVID-19 on social media [71]. Similarly, a simple model constructed from the rate of social media posts can be used as a reliable prediction model when analyzing the uncertainties surrounding the pandemic [72]. Hence, accurate prediction models can be useful tools to model the outbreak of the pandemic as well as in diagnosis prediction [73, 74]. As the fight against the SAR-CoV-2 virus continues, more studies are needed to uncover useful information hidden as a result of the uncertainties surrounding the pandemic.

Despite noteworthy achievements of the existing models, there is a need to indicate how SARS-CoV-2 infection entropy can be applied in predicting the possible number of infections and deaths. In addition, how infection density within a given population contributes to an increase in the number of cases as well as the average time it may take for an infected individual to infect a susceptible population in close proximity. Therefore, the current study is carried out to fill the gap identified above.

## Conclusion and future work

The available COVID-19 vaccines have saved so many lives in the U.S., and beyond. However, several health concerns and uncertainties that have arisen in the wake of the COVID-19 pandemic have yet to be fully resolved. This study presented certain estimation models to determine the possible number of COVID-19 cases and deaths before and after vaccine roll-out in the U.S. The proposed approach shows that a high daily reproduction number for SARS-CoV-2 virus is characterized by an increase in the infection density of the original variant.

This study also shows that COVID-19 infection density can be derived via the Susceptible Infection and Recovered (SIR) model and may be applicable to other infectious diseases such as HIV. The initial behaviour of the SARS-COV-2 virus indicates a high *R*_0_. Such information can be useful in monitoring the behaviour of the virus within a given period as well as in predicting possible future variants. On the projections of the pandemic in late March 2022, using the initial SAR-CoV-2 information entropy of both infection and death growth rates as the determinants for future forecasts. Assuming no vaccines available in the U.S., the current study projects that the number of infections could range between 41,220,199 and 82,440,398, with approximately 1,211,036 deaths.

Furthermore, the current study shows that it takes approximately 7 minutes on average for an infected individual to infect others within a susceptible population in close proximity. Hence, from the initial characteristics of the SAR-CoV-2 virus, this study reports that a single person with COVID-19 can infect approximately 9 people within 1 hour. Consequently, infecting about 216 people in a single day.

The proposed approach can enable other researchers to investigate other unknown factors responsible for the rapid spread of COVID-19, resulting in the emergence of new variants. This study therefore, hypothesize that the new variants (Delta and Omicron) may have a higher daily reproduction number with less daily infection entropy, consequently, spread faster and contagious.

## Data Availability

Not applicable.

## Abbreviations

COVID-19: 2019 novel coronavirus
SARS-CoV-2: Severe acute respiratory syndrome coronavirus 2
WHO: World Health Organization
GISAID: Global Initiative on Sharing All Influenza Data
U.S.: United States
SARS-CoV: Severe acute respiratory syndrome coronavirus
MERS-CoV: Middle East respiratory syndrome coronavirus
GDP: Gross domestic product
SIR: Susceptible Infections and Recovered
IGR: Infection growth rate
SIS: Susceptible-infectious-susceptible
SIRD: Susceptible- infected-recovered-deceased
SARS: Severe Acute Respiratory Syndrome
MERS: Middle East Respiratory Syndrome
HIV: Human immunodeficiency virus
